# Distribution of the c‐MYC gene product in colorectal neoplasia

**DOI:** 10.1111/his.12939

**Published:** 2016-03-17

**Authors:** Ann‐Marie Baker, Susan Van Noorden, Manuel Rodriguez‐Justo, Patrizia Cohen, Nicholas A Wright, Irvin A Lampert

**Affiliations:** ^1^ Centre for Tumour BiologyBarts Cancer InstituteBarts and the London School of Medicine and DentistryQueen Mary University of LondonLondonUK; ^2^ Department of HistopathologyImperial College LondonHammersmith HospitalLondonUK; ^3^ Department of HistopathologyUniversity College LondonLondonUK; ^4^ Department of Cellular PathologyClarence Memorial WingSt Mary's HospitalLondonUK; ^5^ Department of HistopathologyWest Middlesex University HospitalIsleworthUK

**Keywords:** c‐MYC, colorectal cancer, immunohistochemistry, *in‐situ* hybridization

## Abstract

**Aims:**

Recent attempts to study MYC distribution in human samples have been confounded by a lack of agreement in immunohistochemical staining between antibodies targeting the N‐terminus and those targeting the C‐terminus of the MYC protein. The aim of this study was to use a novel *in‐situ* hybridization (ISH) approach to detect *MYC* mRNA in clinically relevant samples, and thereby determine the reliability of MYC‐targeting antibodies.

**Methods and results:**

We performed immunohistochemistry on human formalin‐fixed paraffin embedded normal colon (*n* = 15), hyperplastic polyp (*n* = 4) and neoplastic colon samples (*n* = 55), using the N‐terminally directed antibody Y69, and the C‐terminally directed antibody 9E10. The MYC protein distributions were then compared with the location of *MYC* mRNA, determined by ISH. We found that the localization of *MYC* mRNA correlated well with the protein distribution determined with the N‐terminally directed antibody Y69, and was also associated with expression of the proliferation marker Ki67. The protein distribution determined with the C‐terminally directed antibody 9E10 was not always associated with *MYC* mRNA, Y69, or Ki67, and indeed often showed a reciprocal pattern of expression, with staining being strongest in non‐proliferating cells.

**Conclusions:**

The observed discrepancy between the staining patterns suggests that the significance of 9E10 in immunohistochemical staining is currently uncertain, and therefore should be interpreted with caution.

## Introduction

Cellular MYC (c‐MYC, herein referred to as MYC) plays a role in several fundamental functions of cell biology, including the regulation of cell growth and proliferation, metabolism, differentiation, apoptosis, and angiogenesis.^1^ Not surprisingly, deregulation of MYC is among the most potent activators of tumorigenesis,^2^ and, although *MYC* is the most frequently amplified oncogene in human cancers,^3^ oncogenic mutations in the gene itself are rare. Instead, MYC deregulation is often a result of gene amplification^4^ or mutations upstream of *MYC*; however, in Burkitt's lymphoma, chromosomal translocation is universally the driving event.^5^ One of the important consequences of MYC deregulation is aberrant cellular proliferation, uncontrolled by normal growth factor signalling.

The MYC protein is a transcription factor that comprises an N‐terminal transcription regulatory domain (NTD), a central region containing PEST degradation and nuclear localization signals, and a C‐terminal DNA‐binding domain (CTD). The NTD engages in a variety of protein–protein interactions with components of the transcriptional and chromatin‐remodelling machinery. It contains short sequence motifs (MbI and MbII) that are required for biological activity. The CTD of MYC contains basic helix–loop–helix and leucine zipper (bHLH‐LZ) motifs that mediate interaction with its binding partner MAX and the sequence‐specific DNA binding of the MYC–MAX heterodimer.^3^

One of the common mechanisms of MYC deregulation in cancer is amplification,^6^ so it is amenable to detection by fluorescence *in‐situ* hybridization (FISH), and this can be a highly sensitive technique by which the *MYC* copy number can be studied at cellular resolution. However, there are numerous difficulties associated with performing FISH on formalin‐fixed paraffin‐embedded (FFPE) samples, e.g. the likelihood of experimental artefacts and sample autofluorescence. As it is less prone to these technical problems, antibody‐based detection by immunohistochemistry (IHC) has remained a more widely used and cost‐effective technique. IHC has been shown to provide a good readout of *MYC* amplification, as a correlation between gene amplification determined by *in‐situ* hybridization (ISH) and protein expression determined by IHC has been shown in a number of cancers.^7^, ^8^, ^9^, ^10^, ^11^ Furthermore, IHC can assist in the identification of cases in which protein overexpression has occurred because of chromosomal rearrangement, upstream mutation, or environmental cues.

Several antibodies against MYC have been used for IHC and for western blotting. The ‘gold standard’ was formerly the mouse monoclonal antibody 9E10,^12^ with the target epitope now known to be the C‐terminal 10‐amino acid sequence EQKLISEEDL.^13^ It became clear, however, that 9E10 immunohistochemical results were discrepant with other data, in particular in high‐grade carcinomas, where staining was confined to the tumour cell cytoplasm and the nucleus was negative, whereas it was known that MYC in these instances exerted its function in the nucleus.^14^ Such was the confusion that Williams *et al*.^15^ erroneously posited that MYC had no role in high‐grade carcinomas of the colon.

Recently, antibodies targeting the MYC N‐terminus have been produced: N‐262 (rabbit polyclonal) and Y69 (rabbit monoclonal). Both gave excellent results in prostate^14^ and lymphoid tissue^16^ that were largely consistent with molecular studies. The apparent discrepancy between staining patterns determined by use of the 9E10 antibody with those determined by use of the Y69 antibody or N‐262 antibody has led to uncertainty regarding the localization of MYC in normal and tumour tissue. To determine which of the antibodies reliably detects MYC localization in human samples, we performed ISH for *MYC* mRNA, and compared this with immunohistochemical staining with the C‐terminally targeted 9E10 antibody and the N‐terminally targeted Y69 antibody. As MYC expression is thought to significantly alter during colorectal cancer progression,^17^, ^18^ IHC and ISH were performed on a series of FFPE samples representing normal human colon and a range of colonic neoplasias (*n* = 55).

## Materials and methods

### Patient Samples

FFPE samples were selected from the histopathology archives of Charing Cross and St Mary's Hospitals, London, with the permission of the Imperial College Healthcare Tissue Bank, and from University College Hospital, London, under multicentre ethical approval (07/Q1604/17) according to UK Home Office regulations. All samples were used for IHC, and samples represented by the numbers in square brackets were also used for ISH. Tonsil tissue (*n* = 3[3]) was used to validate our methods. Colon samples consisted of normal tissue (*n* = 15[8]), colorectal adenocarcinomas (*n* = 24[15]), conventional adenomas (*n* = 20[18]), serrated adenomas (*n* = 11[9]), and hyperplastic polyps (HPPs) (*n* = 4[4]). Within the conventional adenomas, there were 13 samples classified as showing low‐grade dysplasia (LGD), six samples showing high‐grade dysplasia (HGD), and one sample showing both LGD and HGD. Adenomas were classified by two expert pathologists, according to the recommendations of the Bowel Cancer Screening Programme Pathology Group (2007) and European Guidelines.^19^

### Immunohistochemistry

Details of immunohistochemical methods and absorption studies are given in the Supporting information.

### *In‐Situ* Hybridization

ISH for *MYC* mRNA expression was performed on 5‐μm sections with the RNAscope 2.0 High Definition assay (310036; Advanced Cell Diagnostics, Hayward, CA, USA), as previously described.^20^ The RNAscope probes used were *MYC* (NM_002467.4, region 536–1995, catalogue number 311761), *PPIB* (positive control probe, NM_000937.4, region 139–989, catalogue number 313901), and *dapB* (negative control probe, EF191515, region 414–862, catalogue number 310043).

## Results

### MYC Distribution in Human Tonsil

We initially examined human tonsil lymphoid tissue (*n* = 3) in order to confirm that our immunohistochemical staining was consistent with that previously published. In general, immunostaining patterns in the tonsil agreed with those of Cattoretti,^16^ showing large Y69‐positive cells in the light zone and the periphery of the dark zone (founder cells), and smaller numbers in the mantle zone (Figure S1). 9E10‐positive cells followed this pattern, with additional positivity as compared with Y69. ISH for *MYC* mRNA, which has not previously been performed, showed a wider distribution of *MYC* expression than that shown by MYC IHC (Figure S1), suggesting post‐transcriptional regulation of MYC.

### MYC Distribution in Normal and Neoplastic Human Colon

**Figure 1:**
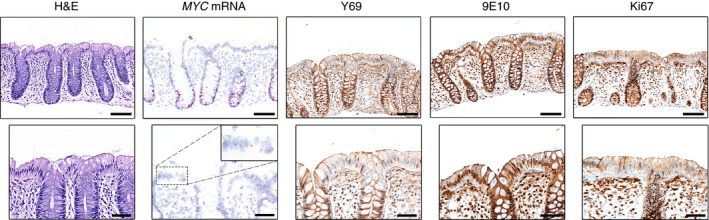
MYC expression in normal human colon. Representative haematoxylin and eosin staining, *in‐situ* hybridization (*MYC* mRNA, pink) and immunohistochemical staining with Y69 (N‐terminal MYC), 9E10 (C‐terminal MYC) and Ki67 antibodies (brown) in normal human colon. The lower panels focus on the surface epithelium, which appears negative for Y69 and Ki67 expression, but strongly positive for 9E10 expression. Scale bars: 100 μm (upper panels) and 50 μm (lower panels).

We next examined morphologically normal human colon (*n* = 15) and noted that, as previously documented,^21^ the lower third of the crypt showed positive staining for Y69 (Figure 1). As MYC expression is associated with proliferation, we also examined Ki67 expression, and found it to be consistent with Y69 staining (Figure 1). The ISH for *MYC* mRNA was visualized as cytoplasmic and nuclear red dots of variable size, which were sometimes fused to form larger foci of staining (Figure S2). We found that *MYC* mRNA expression was associated with Y69 and Ki67 staining; however staining with the 9E10 antibody did not correlate with the other markers.

The nuclei of the luminal surface epithelium were generally negative for Y69 and Ki67. Nevertheless, we noted the presence of *MYC* mRNA in cells of the surface epithelium, suggesting that a low level of MYC expression may have been present (Figure 1). In contrast to the other markers, 9E10 staining was strong in the nuclei of most of the surface epithelial lining cells, and extended to the basal portion of the crypts (Figure 1). It is also notable that, in the lamina propria, most of the lymphocytes were 9E10‐positive and only rarely positive for Y69 or *MYC* mRNA.

Because 9E10 reactivity differed from that of the other MYC markers and was in the non‐proliferating surface cells, we confirmed the antibody specificity by preabsorbing it with its specific epitope peptide. In both normal colon (Figure S3A) and tumour tissue (Figure S3B), staining was abolished by preincubation of the antibody with 0.625–6.25 μg/ml of the blocking peptide, whereas 10 μg/ml of a peptide of a similar length, cerebellin, had no effect. As a further control, we noted that 12.5 μg/ml of the blocking peptide had no effect on Y69 staining (Figure S3C).

**Figure 2:**
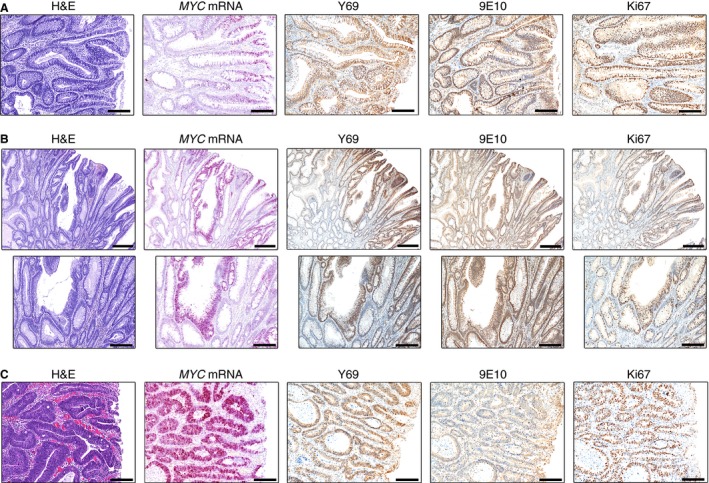
MYC expression in conventional adenomas. **A**, Representative haematoxylin and eosin (H&E) staining, *in‐situ* hybridization (*MYC* mRNA, pink) and immunohistochemical staining with Y69 (N‐terminal MYC), 9E10 (C‐terminal MYC) and Ki67 antibodies (brown) in adenoma with low‐grade dysplasia. A ‘reverse gradient’ of *MYC* mRNA and Y69 expression, with highest expression at the luminal surface, is apparent. Scale bars: 200 μm. **B**, Representative H&E staining, *in‐situ* hybridization (*MYC* mRNA, pink) and immunohistochemical staining with Y69 (N‐terminal MYC), 9E10 (C‐terminal MYC) and Ki67 antibodies (brown) in adenoma, focusing on a gland with a highly proliferative phenotype. Again, a reverse gradient is apparent, although, in the proliferative gland, the *MYC* mRNA, Y69 and Ki67 expression can be seen extending to the base of the crypt, in contrast to the surrounding glands and normal crypts. Scale bars: 500 μm (upper panels) and 200 μm (lower panels). **C**, Representative H&E staining, *in‐situ* hybridization (*MYC* mRNA, pink) and immunohistochemical staining with Y69 (N‐terminal MYC), 9E10 (C‐terminal MYC) and Ki67 antibodies (brown) in high‐grade dysplasia. Extensive positivity for *MYC* mRNA, Y69 and Ki67 is apparent, whereas 9E10 staining is remarkably weak. Scale bars: 200 μm.

In conventional adenomas with LGD (*n* = 14), *MYC* mRNA showed the greatest density in the surface epithelium near the luminal orifice of the glands (Figure 2A), with expression decreasing towards the base of the glands. The staining pattern of Y69 was in agreement with this. We noted the presence of adenomatous glands that showed a highly proliferative phenotype. In these glands, the expression of *MYC* mRNA and Y69 extended through the length of the gland, and was no longer restricted to the surface (Figure 2B). In regions of HGD (*n* = 7), we found that all glands showed very high levels of *MYC* mRNA and Y69 expression (Figure 2C). Strikingly, in all adenoma samples, we again found that 9E10 positivity showed the reverse distribution to the other markers, in that the extent of 9E10 positivity in the tubules was less in HGD regions than in LGD regions, and, indeed, the area of the tubules stained by 9E10 appeared to be inversely related to the severity of the grading (Figure 2).

Cribriform architecture, which is characteristic of severe dysplasia, was seen in several specimens. This disorganization is reflected in the location of the *MYC* mRNA and of Y69 immunostaining (Figure S4). In the instances studied, the nuclei were negative for 9E10, again in contrast to the expression of the other markers.

Serrated lesions have a distinct pathway of progression, and we have recently shown that serrated adenomas retain a stem cell hierarchy, whereas their conventional counterparts do not.^20^ We therefore examined MYC expression in traditional serrated adenomas (TSAs) (*n* = 3), sessile serrated adenomas (SSAs)/sessile serrated polyps (SSPs) (*n* = 6), and HPPs (*n* = 4).

**Figure 3:**
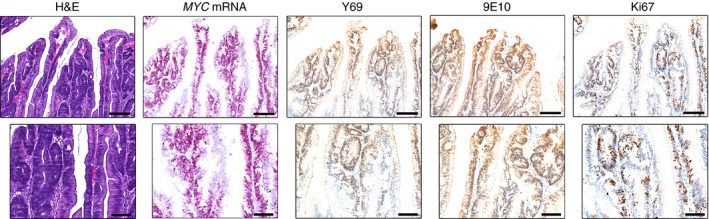
MYC expression in traditional serrated adenomas. Representative haematoxylin and eosin staining, *in‐situ* hybridization (*MYC* mRNA, pink) and immunohistochemical staining with Y69 (N‐terminal MYC), 9E10 (C‐terminal MYC) and Ki67 antibodies (brown) in a traditional serrated adenoma. Strong positivity for *MYC* mRNA, Y69 and Ki67 is apparent in ectopic crypt foci. Scale bars: 200 μm (upper panels) and 100 μm (lower panels).

TSAs showed irregular foci of growth perpendicular to the line of the crypt (‘ectopic crypts’). In the bases of these ectopic foci, positive staining for *MYC* mRNA, Y69 and Ki67 was seen (Figure 3). 9E10 staining was moderate throughout the lesion, but the marked high intensity of expression of the other markers at the bases of ectopic crypts was not seen (Figure 3).

**Figure 4:**
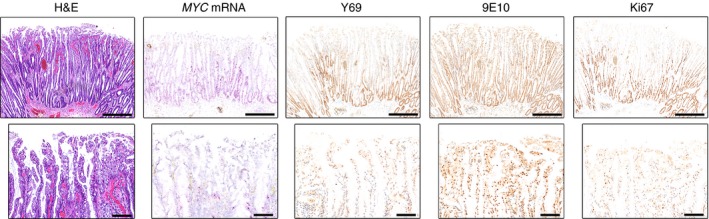
MYC expression in hyperplastic polyps. Representative haematoxylin and eosin staining, *in‐situ* hybridization (*MYC* mRNA, pink) and immunohistochemical staining with Y69 (N‐terminal MYC), 9E10 (C‐terminal MYC) and Ki67 antibodies (brown) in a hyperplastic polyp. The lower panels focus on the surface epithelium, which appears negative for Y69 and Ki67 expression, but strongly positive for 9E10 expression. Scale bars: 500 μm (upper panels) and 100 μm (lower panels).

We next examined the MYC distribution in HPPs, which are traditionally regarded as benign serrated lesions. In HPPs, the distribution of the markers was similar to that seen in the normal colon, with Ki67, Y69 and *MYC* mRNA concentrated in the basal portions of the crypts (Figure 4). As previously reported,^20^, ^22^ this zone of proliferation is more extensive than that of normal colon, but does not extend to the surface epithelium. However, in contrast to the other markers, we observed high levels of 9E10 staining in the surface epithelium (Figure 4). The staining observed in SSA/SSPs was generally similar to that described for HPPs.

**Figure 5:**
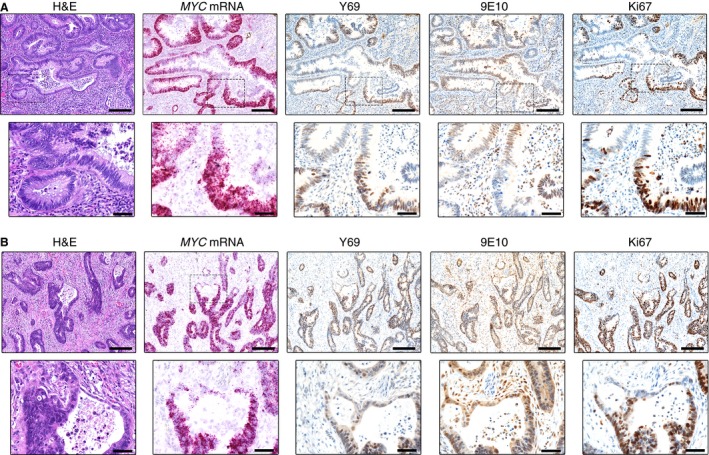
MYC expression in invasive carcinoma. **A**, Representative haematoxylin and eosin (H&E) staining, *in‐situ* hybridization (*MYC* mRNA, pink) and immunohistochemical staining with Y69 (N‐terminal MYC), 9E10 (C‐terminal MYC) and Ki67 antibodies (brown) in a region of invasive carcinoma. The lower panels highlight the reciprocal relationship between the expression of *MYC* mRNA, Y69, and Ki67, and the staining of the 9E10 antibody. **B**, Representative H&E staining, *in‐situ* hybridization (*MYC* mRNA, pink) and immunohistochemical staining with Y69 (N‐terminal MYC), 9E10 (C‐terminal MYC) and Ki67 antibodies (brown) in a region of invasive carcinoma. The lower panels focus on an invasive gland showing flattened non‐proliferative cells at the invasive edge, which are notably Y69‐negative and 9E10‐positive. Scale bars: 200 μm (upper panels) and 50 μm (lower panels).

In invasive carcinomas, the expression of MYC protein (by Y69 staining) and *MYC* mRNA was much greater than in most of the adenomas, but similar to that in those with the most severe dysplasia (Figure 5A,B). We noted a high level of heterogeneity in invasive glands, some of which contained regions of low MYC expression and, indeed, negative areas (Figure 5A). As previously reported,^15^ 9E10 nuclear staining was often absent in carcinomas. However, there were frequent scattered stretches of neoplastic epithelium that were positive for 9E10, but negative for Y69 and Ki67, and with low levels of *MYC* mRNA. We noted examples of such areas adjacent to focal regions of proliferating cells; the reciprocal relationship between Y69 and 9E10 staining was particularly clear here (Figure 5A).

Rubio^23^ described flattened areas in the advancing edge of colonic adenocarcinomas that were negative for Ki67. Our results confirm this (Figure 5B), and we further note that Y69 immunopositivity and *MYC* mRNA were also absent. However, these nuclei were positive for 9E10. Additional foci of stromal invasion could be found that were not flattened, and many of these showed the same distribution of markers.

## Discussion

MYC is accepted as one of the most potent drivers of tumorigenesis; however, recent attempts to examine its expression have been confounded by inconsistencies in antibody immunoreactivity. We have attempted to resolve this issue by comparing detection of MYC by the use of conventional IHC with a novel ISH approach that specifically detects *MYC* mRNA in FFPE tissue sections.

We have shown that *MYC* mRNA, MYC protein (by Y69 immunostaining) and Ki67 are found in the lower third of normal human colonic crypts. However, we have found that positivity of the C‐terminally targeted antibody 9E10 does not always correlate with these markers. In fact, the staining often shows a striking inverse relationship, with the non‐proliferating surface epithelium of normal crypts staining positively for 9E10. There are very low, but detectable, levels of *MYC* mRNA in the surface epithelium, and MYC expression has been shown in non‐proliferating cells.^24^, ^25^, ^26^ Therefore, 9E10 could be detecting an alternative conformation of MYC, which may not play a role in driving proliferation. For example, there is a form of MYC (‘MYC‐S’) that lacks the proximal 100 N‐terminal amino acids,^27^ containing the region in which the Y69 epitope is located. However MYC‐S has been shown to retain the ability to stimulate proliferation,^28^ so it probably cannot explain the 9E10 staining in the non‐proliferating cells of the surface epithelium. Alternatively, although our absorption studies have shown the staining to be specific to the epitope, it is possible that 9E10 may cross‐react with another protein.

As MYC plays a role at some stage in the development of most colorectal carcinomas,^18^ we studied the spatial distribution of MYC protein and *MYC* mRNA in adenomas. We concluded that the distribution of these markers agreed remarkably well with the traditional morphological classification. Our results suggest strong expression of *MYC* mRNA and Y69 in high‐grade lesions and in invasive carcinomas. We confirm the results of a previous report^23^ that described flattened Ki67‐negative cells at the invading edge of colorectal carcinomas. We found this feature only as a focal occurrence, but confirm that, in such areas, both MYC expression and Ki67 are down‐regulated. Such changes in cell shape and gene expression could be indicative of an epithelial‐to‐mesenchymal transition, which is known to be a feature of the invasive phenotype.^29^

Cribriform architecture in adenomatous glands indicates HGD. Here, the highly irregular localization of *MYC* mRNA suggests that there is disorientation of cell growth, a marker of malignancy. The intense expression of MYC in the carcinomas is in line with the known overproduction of *MYC* mRNA and MYC protein described in these processes.^18^ As in all areas of high‐grade malignancy, 9E10 staining was notably absent. The absence of 9E10 staining in high‐grade neoplastic cells has been previously reported.^14^, ^15^ Suggested explanations include deletion of parts of the MYC molecule, the presence of unknown isomers, and interference with 9E10 binding by other nuclear proteins.

Another explanation for the apparent reciprocal relationship between Y69 and 9E10 immunoreactivity relates to the physical nature of the MYC molecule. MYC is one of a group of so‐called intrinsically disordered proteins,^30^, ^31^ and can adopt conformations ranging from collapsed to fully extended. Both the unstructured NTD and the largely unstructured bHLH‐LZ domain at the C‐terminus change their configurations when bound to certain proteins. It has been shown that monoclonal antibody binding is affected by structural changes in proteins,^32^, ^33^ so it is possible that structural changes in MYC can affect the binding of anti‐MYC antibodies.

In this study, we have used a comparison of ISH and IHC to raise key questions concerning the specificity of the MYC‐targeting antibody 9E10. Our results suggest that 9E10 does not recognize MYC in its classic proliferation‐associated form; however, this does not exclude the possibility that it binds to a different conformation of MYC. As 9E10 positivity occurs when proliferation appears to be inhibited, it follows that MYC may adopt different conformations to promote contradictory cellular states, a possibility that is suggested by experimental inhibitors of the HUWE1 ubiquitin ligase.^34^ Further investigation into the specificity of MYC‐targeting antibodies is needed, and care should be taken when drawing conclusions from immunohistochemical staining for MYC, particularly with 9E10.

## Conflicts of interest

The authors declare no conflict of interest.

## Author contributions

Experiments were designed and conceived by I. A. Lampert and S. Van Noorden, and performed by A.‐M. Baker and S. Van Noorden. The manuscript was written by A.‐M. Baker, S. Van Noorden, and I. A. Lampert, with assistance from M. Rodriguez‐Justo and N. A. Wright. M. Rodriguez‐Justo and P. Cohen carried out the histopathology diagnosis and grading. N. A. Wright provided essential guidance and critical analysis throughout the process.

## Supporting information


**Figure S1.** MYC expression in human tonsil.

**Figure S2.** *MYC* mRNA ISH in normal human colon.

**Figure S3.** Absorption studies using the 9E10 blocking peptide EQKLISEEDL.

**Figure S4.** MYC expression in high‐grade dysplasia.
