# Supplementary figures and images for: Distribution of the c‐MYC gene product in colorectal neoplasia
Source: Histopathology. 2016 Mar 17;69(2):222–9. doi: 10.1111/his.12939 (PMC4949543; doi:10.1111/his.12939)

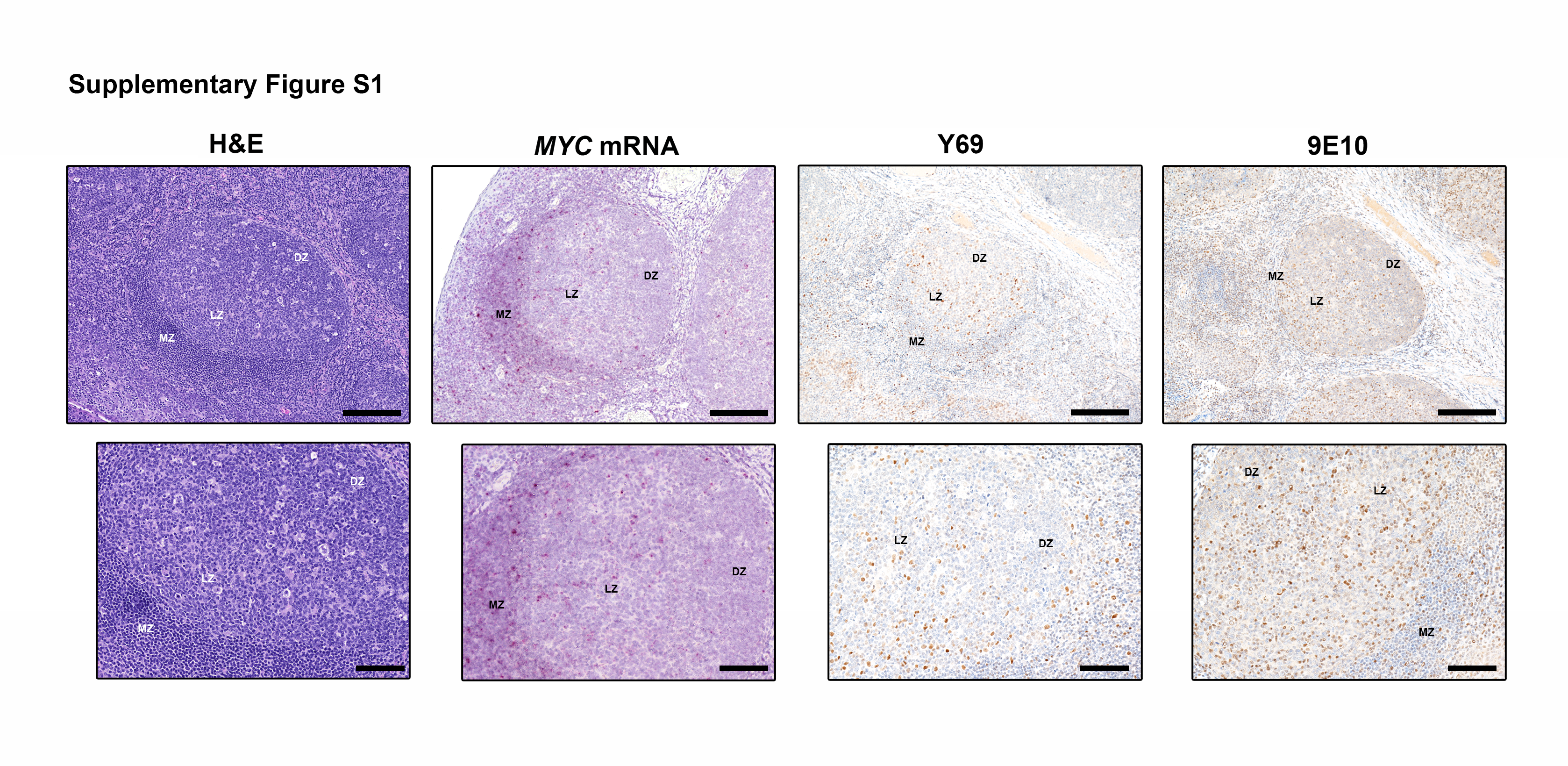

Supplement: Supplementary file 1 — Figure S1. MYC expression in human tonsil. [file HIS-69-222-s001.tiff]

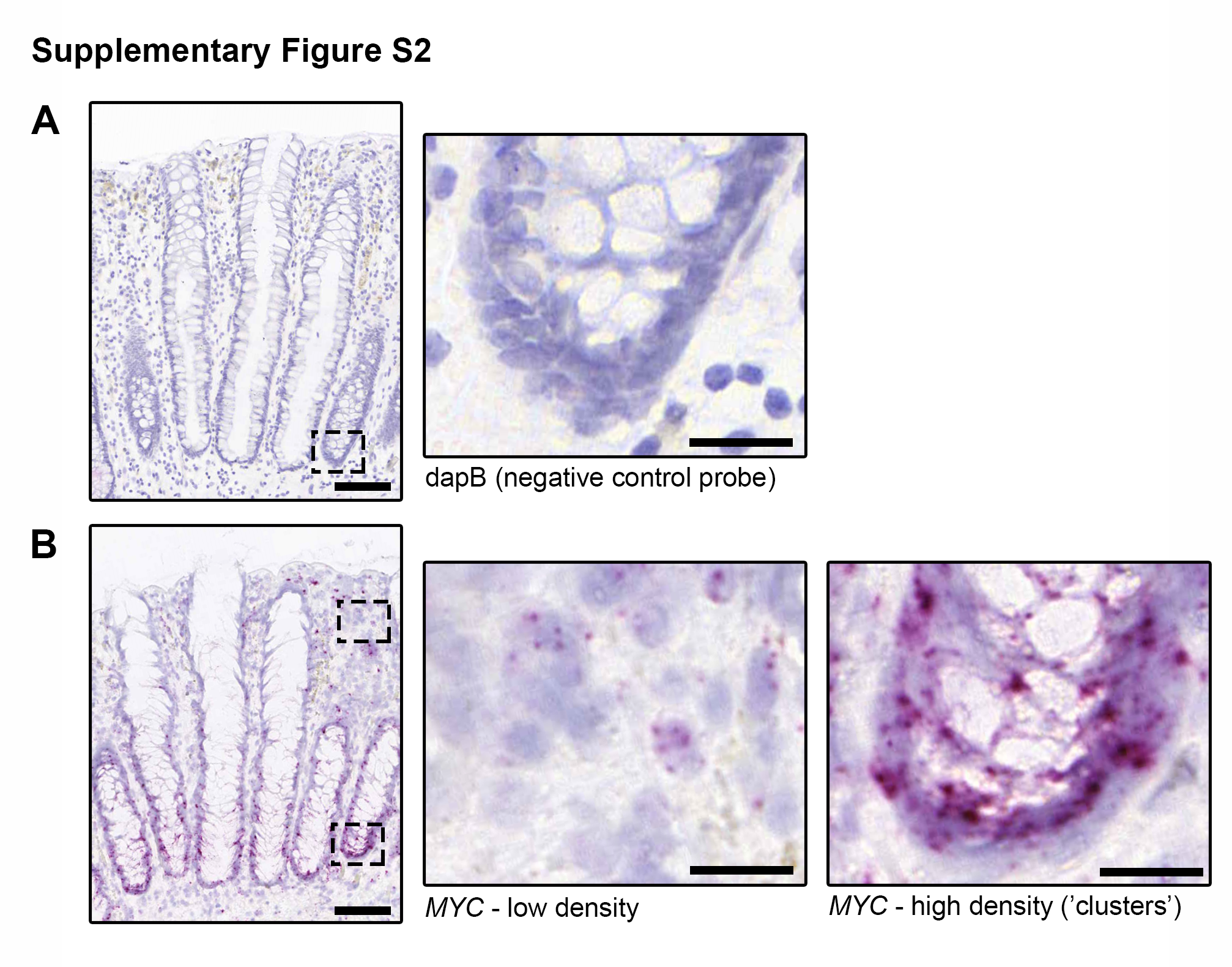

Supplement: Supplementary file 2 — Figure S2. MYC mRNA ISH in normal human colon. [file HIS-69-222-s002.tiff]

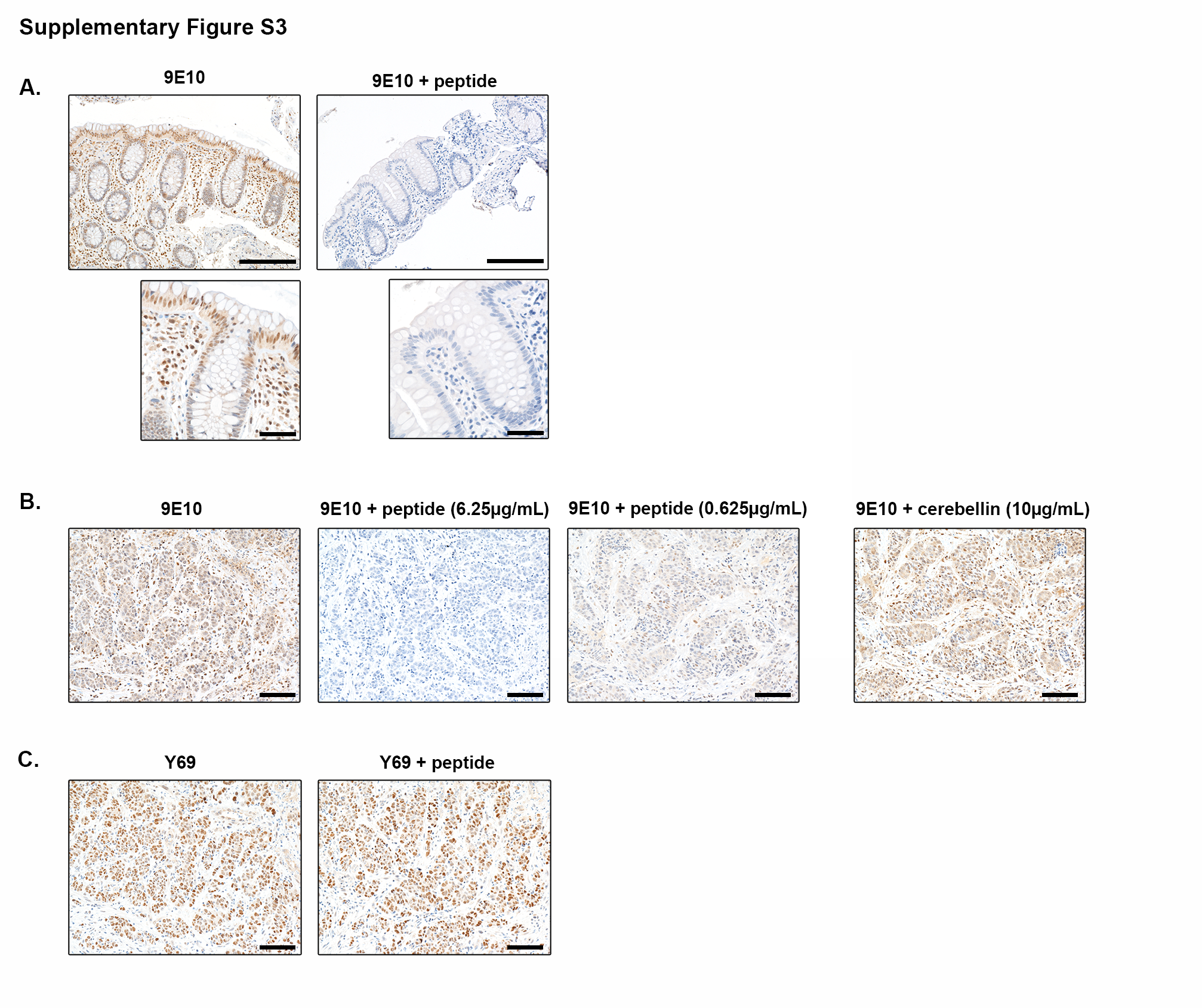

Supplement: Supplementary file 3 — Figure S3. Absorption studies using the 9E10 blocking peptide EQKLISEEDL. [file HIS-69-222-s003.tiff]

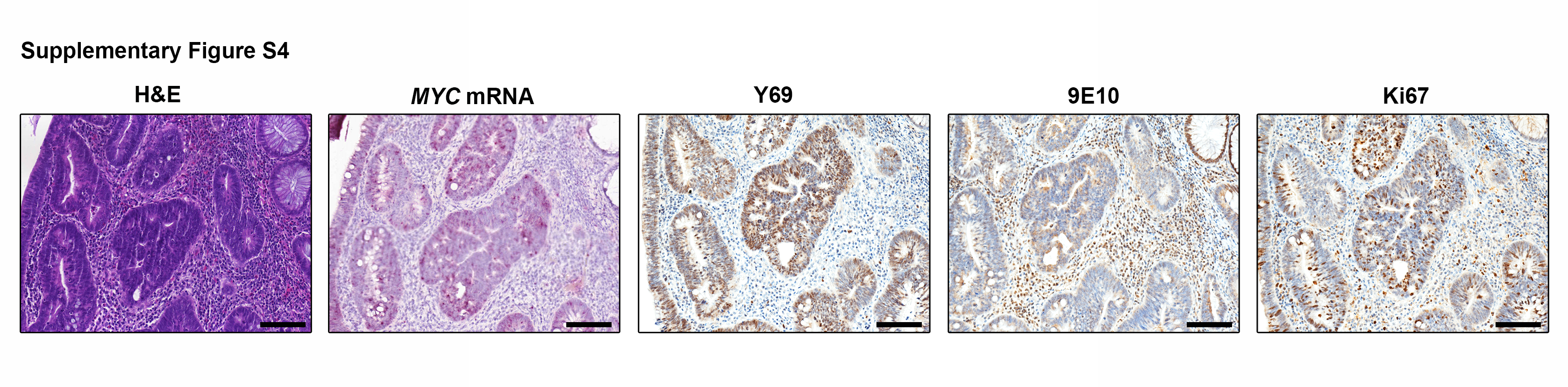

Supplement: Supplementary file 4 — Figure S4. MYC expression in high‐grade dysplasia. [file HIS-69-222-s004.tiff]
