# Supplementary material for: Distribution of the c‐MYC gene product in colorectal neoplasia
Source: Histopathology. 2016 Mar 17;69(2):222–9. doi: 10.1111/his.12939 (PMC4949543; doi:10.1111/his.12939)
Supplement: Supplementary file 5 [file HIS-69-222-s005.docx]

**Supplementary Figure Legends**

**Supplementary Figure S1. MYC expression in human tonsil**

Representative H&E staining, *in situ* hybridisation (*MYC* mRNA, pink) and immunohistochemical staining using Y69 (N-terminal MYC) and 9E10 (C-terminal MYC) antibodies in human lymphoid tonsil issue. The ISH shows many positive cells in the mantle zone and scattered positive cells in the dark and light zones. IHC with Y69 shows positive nuclei predominantly in the germinal centre and scattered in the mantle zone. IHC with 9E10 shows scattered positive cells in the dark and light zones. MZ = mantle zone, LZ = light zone, DZ = dark zone. Scale bars represent 200 micron or 100 micron (inset).

**Supplementary Figure S2. *MYC* mRNA ISH in normal human colon**

**A.** Representative *in situ* hybridisation in the normal colon using the negative control probe dapB.

**B.** Representative *in situ* hybridisation for *MYC* mRNA in the normal colon. *MYC* mRNA is present at low density at the luminal surface of the crypts, and at high density at the crypt base, where the staining forms clusters of positivity. Scale bars in A and B represent 100 micron and 20 micron (inset).

**Supplementary Figure S3. Absorption studies using the 9E10 blocking peptide EQKLISEEDL**

**A.** Normal colon stained with the 9E10 antibody (left panel) and with the 9El0 antibody pre-incubated with the EQKLISEEDL peptide at 6.25µg/mL (right panel). Pre-absorption with the peptide clearly removes all staining. Scale bars represent 200 micron (upper panels) and 50 micron (lower panels).

**B.** Colon carcinoma stained with 9E10 antibody pre-incubated with decreasing concentrations of the EQKLISEEDL peptide. There is a clear dose-dependent reduction in staining intensity. Pre-incubation of 9E10 with the cerebellin peptide has no effect on staining.

**C.** Colon carcinoma stained with Y69 antibody (left panel) and Y69 antibody pre-incubated with the EQKLISEEDL peptide at 12µg/mL (right panel). The peptide has no effect on Y69 staining. Scale bars in B and C represent 100 micron.

**Supplementary Figure S4. MYC expression in high-grade dysplasia**

Representative H&E staining, *in situ* hybridisation (*MYC* mRNA, pink) and immunohistochemical staining using Y69 (N-terminal MYC), 9E10 (C-terminal MYC) and Ki67 antibodies (brown) in a region of high-grade dysplasia, displaying cribriform architecture. *MYC* mRNA, Y69 and Ki67 expression are generally high, but 9E10 staining is notably low. Scale bars represent 100 micron.
